# Engagement and Participant Experiences With Consumer Smartwatches for Health Research: Longitudinal, Observational Feasibility Study

**DOI:** 10.2196/14368

**Published:** 2020-01-29

**Authors:** Anna L Beukenhorst, Kelly Howells, Louise Cook, John McBeth, Terence W O'Neill, Matthew J Parkes, Caroline Sanders, Jamie C Sergeant, Katy S Weihrich, William G Dixon

**Affiliations:** 1 Centre for Epidemiology Versus Arthritis, Centre for Musculoskeletal Research University of Manchester Manchester Academic Health Science Centre Manchester United Kingdom; 2 The National Institute for Health Research, School for Primary Care Research University of Manchester Manchester Academic Health Science Centre Manchester United Kingdom; 3 National Institute for Health Research Manchester Biomedical Research Centre Manchester University National Health Service Foundation Trust Manchester Academic Health Science Centre Manchester United Kingdom; 4 Department of Rheumatology Salford Royal National Health Service Foundation Trust Salford United Kingdom; 5 National Institute for Health Research Greater Manchester Patient Safety Translational Research Centre University of Manchester Manchester United Kingdom; 6 Centre for Biostatistics University of Manchester Manchester Academic Health Science Centre Manchester United Kingdom

**Keywords:** medical informatics computing, mHealth, patient-reported outcomes, musculoskeletal diseases, mobile phone, smartwatch/wearable, self-tracking

## Abstract

**Background:**

Wearables provide opportunities for frequent health data collection and symptom monitoring. The feasibility of using consumer cellular smartwatches to provide information both on symptoms and contemporary sensor data has not yet been investigated.

**Objective:**

This study aimed to investigate the feasibility and acceptability of using cellular smartwatches to capture multiple patient-reported outcomes per day alongside continuous physical activity data over a 3-month period in people living with knee osteoarthritis (OA).

**Methods:**

For the KOALAP (Knee OsteoArthritis: Linking Activity and Pain) study, a novel cellular smartwatch app for health data collection was developed. Participants (age ≥50 years; self-diagnosed knee OA) received a smartwatch (Huawei Watch 2) with the KOALAP app. When worn, the watch collected sensor data and prompted participants to self-report outcomes multiple times per day. Participants were invited for a baseline and follow-up interview to discuss their motivations and experiences. Engagement with the watch was measured using daily watch wear time and the percentage completion of watch questions. Interview transcripts were analyzed using grounded thematic analysis.

**Results:**

A total of 26 people participated in the study. Good use and engagement were observed over 3 months: most participants wore the watch on 75% (68/90) of days or more, for a median of 11 hours. The number of active participants declined over the study duration, especially in the final week. Among participants who remained active, neither watch time nor question completion percentage declined over time. Participants were mainly motivated to learn about their symptoms and enjoyed the self-tracking aspects of the watch. Barriers to full engagement were battery life limitations, technical problems, and unfulfilled expectations of the watch. Participants reported that they would have liked to report symptoms more than 4 or 5 times per day.

**Conclusions:**

This study shows that capture of patient-reported outcomes multiple times per day with linked sensor data from a smartwatch is feasible over at least a 3-month period.

**International Registered Report Identifier (IRRID):**

RR2-10.2196/10238

## Introduction

### Background

Wearables, such as activity trackers, provide opportunities for frequent monitoring of chronic diseases. Their sensors can record behaviors of interest at high temporal and spatial resolution [1-4]. Wearables are widely used: in 2016 there were 325 million connected wearable devices worldwide, with half of the owners wearing their device every day [5,6]. In health care, sensor data from wearables would be even more relevant if combined with simultaneously collected patient-reported outcomes. This would enable symptom monitoring, adding context to the sensor outputs, and may aid clinical decision making and empower patients [7].

### Consumer Cellular Smartwatches

A new technical innovation enables collection of sensor data alongside patient-reported outcomes. In 2017, the first *cellular* smartwatches came to market. Cellular smartwatches combine the functionalities of smartphones (touch screen, SIM card and cellular connection, and possibility to develop and install apps) with wearables (passive collection of sensor data, wrist-worn). This enables frequent collection of patient-reported outcomes (via touchscreen) alongside accurate and objective information on behavior or exposure (from sensors). Furthermore, these data can be collected in real time and automatically uploaded to remote servers without the need for pairing with a smartphone or another device for connectivity.

### Physical Activity and Knee Osteoarthritis

An example of a clinical disease area where the pairing of symptoms and sensor data, especially on physical activity, could significantly advance research is arthritis [8]. Knee osteoarthritis (OA) is one of the most common types of arthritis: it affects 19% to 28% of men and women older than 45 years and is characterized by disabling knee pain and a reduction in mobility [9]. Physical activity is beneficial in reducing long-term pain severity and disability [10] and has cardiovascular and other benefits. However, the relationship between pain and activity is complex: pain can limit the amount of physical activity that is possible, while increasing physical activity beyond a certain level may further increase pain severity [11]. Wearable devices have been used to track physical activity in OA research [12], but frequent symptoms are rarely collected in parallel. Furthermore, proprietary algorithms from consumer fitness trackers are less accurate in arthritis patients because gait characteristics differ between healthy people and those with musculoskeletal conditions [13]. Understanding the interplay between physical exercise and symptoms would be an important step in helping to develop and target personalized interventions to support an appropriate level of physical activity. For example, encouraging more physical activity within an individual’s personal threshold. This requires frequent, accurate, and granular data on pain symptoms and activity, which cellular smartwatches may be able to provide.

### Objectives

The feasibility of collecting such data through cellular smartwatches remains uncertain. Knowledge of barriers and enablers of engaging with cellular smartwatches long term could inform the design of future studies. To address this, we conducted the KOALAP (Knee OsteoArthritis: Linking Activity and Pain) study. We developed a cellular smartwatch app for collection of patient-reported outcomes (multiple times a day) alongside continuous sensor data. The aim of this feasibility study was to investigate engagement patterns and acceptability of collecting health and behavior data using consumer cellular smartwatches daily for 3 months. Specifically, the study objectives were to report participant engagement, to investigate participant views and experiences, and to identify barriers and enablers to collecting data through cellular smartwatches.

## Methods

### Subjects and Data Collection From the Smartwatch

Men and women older than 50 years with self-reported knee OA were recruited in September 2017 for participation in a 90-day observational study. Detailed methods have been reported elsewhere [14].

**Figure 1:**
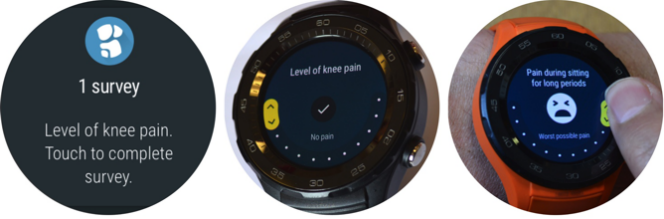
User interface of KOALAP app. Left: notification of an active survey; middle: data entry screen for survey “level of knee pain”; right: data are entered by swiping the numeric rating scale icon.

In brief, participants received a Huawei Watch 2 preinstalled with the KOALAP study app (all other features and apps were disabled) developed by the study team and Google Android Wear. Participants were instructed to wear the watches for 90 days, from waking until going to bed, and answer the watch questions when prompted (Figure 1). At baseline, participants reported age, gender, and previous experience with health technology (see [14]), and, for the watch questions, the activity that caused most knee pain and the activity that was most important for them to do without knee pain. At study completion, participants received a Web-based questionnaire with questions about their experiences with the watch, for example, “I often forgot to charge the watch or wear it again after charging.” The full questionnaire is available as an appendix to the published study protocol [14].

The KOALAP app triggered 4 or 5 questions on knee pain and quality of life per day. These questions had to be answered within a specific time window, which took around 10 seconds per question. The questions were:

- Level of knee pain (twice-daily at 12.22 and 18.22; window 4 hours)
- Knee pain affecting daily activities (daily at 17.00; window 7 hours)
- Knee pain during painful activity (as reported at baseline); daily at 17.00; window 12 hours)
- Pain interference with important activity (as reported at baseline); weekly on Wednesday at 12.00; window 12 hours)
- Impact of knee symptoms on quality of life (weekly on Sunday at 12.00; window 12 hours)
- 17 questions from the Knee Injury and Osteoarthritis Outcome Score [14,15] (monthly; window 7 days)

When a participant was wearing the watch, the KOALAP app collected raw sensor data. When participants took off the watch, *off-body detection* stopped sensor data collection. On the home screen of the watch, participants could see their heart rate and step count as calculated by the Android operating system. During recharging, data were uploaded to the study servers and deleted from the watch. If participants were abroad or had poor cellular signal at the charging location, the data upload failed, and the watch stopped collecting sensor data.

### Participant Interviews

All participants were invited to take part in 2 separate interviews (1 shortly after baseline and 1 on completion of the study). A semistructured interview schedule (see Multimedia Appendix 1) was developed from the sociological research literature on self-tracking [16-21]. This literature is split between a techno-utopian approach and a critical approach. The techno-utopian approach suggests that self-tracking can empower and motivate individuals to adopt a healthy lifestyle. The critical approach has focused more on the implications of self-tracking for privacy, personal responsibility, surveillance, and changing views of the body and health. The interview schedule was also informed by factors known to affect attrition in digital health studies, including usability, feedback, perceived advantages of participation, time required, user experience, and external events such as health [22]. The interviews at baseline explored participants’ experiences of living with OA, motivations and expectations of using a smartwatch, and previous experiences with health technology. We were interested if previous engagement with devices had changed health-related behaviors. At follow-up, interviews explored participants’ experiences of using the watch and being monitored and whether the knowledge gained from using the watch (if any) had significant implications for their understanding of knee OA.

### Analysis

### Engagement With Smartwatch

The primary measures of engagement were the number of active participants per day, hours of wear time, and completeness of watch questions. Active participants were defined as participants who wore the watch for at least 30 min. Wear time per participant-day was defined as the total hours of available sensor data, rounded to the nearest hour. The completeness of watch questions was defined as the percentage of watch questions completed (per specific watch question over the study duration; per participant-day). For each study day, we calculated mean wear time and mean completeness of watch questions across all participants and across all active participants.

In addition, we determined temporary and permanent nonusage attrition and the mean clock time that participants put on and took off the watch. Temporary nonusage attrition refers to the participants that are not active for a period (ie, do not wear the watch for >30 min but later resume wearing the watch). Permanent nonusage attrition refers to participants that are not active and never again wear the watch [22]. Per participant over the study period, we determined the average clock time of the first and last sensor data record. When participants took off and put on the watch multiple times, we only considered the longest continuous episode per day for calculating these clock times.

#### End-of-Study Survey: Participant Experiences

Descriptive statistics were used to summarize responses to the baseline and end-of-study surveys.

#### Participant Interviews

Interviews were audio-recorded and transcribed verbatim and coded using NVIVO (QSR International). Transcripts were analyzed thematically, drawing on some of the key techniques of grounded theory [23], including open coding, constant comparison, and memo writing**.** Verbatim quotes that illustrate the key themes were selected.

#### Case Studies

To illustrate how interview themes relate to individual levels of engagement, 2 case studies of participants were analyzed, combining quantitative engagement data with interview quotes.

This study underwent full review by the University of Manchester Research Ethics Committee (#0165) and University Information Governance (#IGRR000060).

## Results

### Subjects and Baseline Survey

A total of 26 subjects took part in the study. Their mean age was 64 years, and 50% (13/26) were female (13/26). Before enrollment, 9 participants had used a smartphone only (n=3), wearable only (n=3), or both (n=3) for health or activity monitoring.

**Figure 2:**
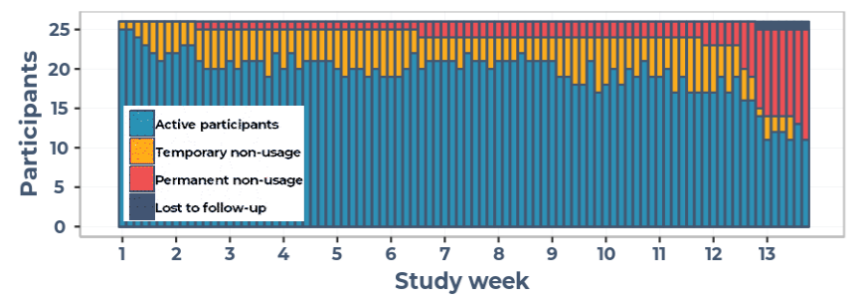
Active participants (blue) per study day, participants temporary nonusage (yellow), permanent nonusage (red) or lost to follow-up (dark blue).

In total, 6894 watch questions and 643 gigabytes of sensor data were received over the 90-day study period. Participants wore the watch on 73% (81/90) of days. Over time, the number of active participants decreased (Figure 2): from 25 on the first day to 11 on the last day. Until the last study week, the main form of attrition was temporary nonusage attrition (participants not wearing the watch but later re-engaging). Permanent nonusage attrition (participants not wearing the watch again during the study) was low in the first 2 months: 1 participant stopped in the first month and 1 in the second month. In the last study month, 13 participants stopped using the watch (of which 8 in the last week), of which 1 was lost to follow-up after day 84 (ie, did not fill in the end-of-study-questionnaire and did not return the watch).

### Engagement With Smartwatch

**Figure 3:**
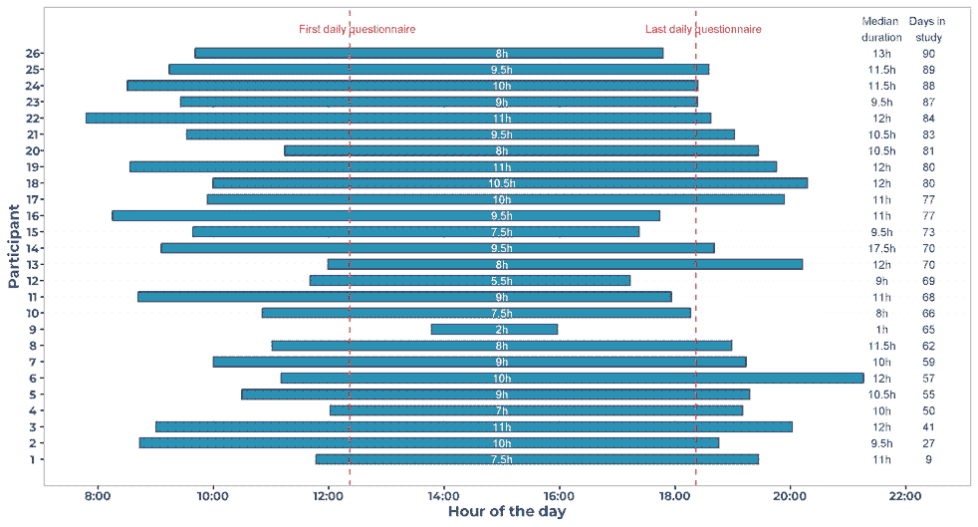
Average duration of continuous sensor data collection, per participant. Each bar corresponds to a participant. The bar starts at the average time of day that sensor data collection started and ends at the average time of day that sensor data collection ceased (duration in hours shown in middle of bar). On the right: the median wear time and number of days the participant was active. Median wear time can be higher than clock time duration of the longest wearing episode, as some participants recharged the watch and put it on multiple times.

The median daily wear time among active participants was 11 hours 12 min (interquartile range 9 hours 27 min-12 hours 6 min). The mean time-of-day at which sensor data collection started and stopped varied between participants: from 07.48 to 13.48 and 16.00 and 21.18, respectively (Figure 3). For most participants, this covered the trigger time for all watch questions from first (12.22) to the last (18.22). For 1 participant, average wear time started after the first trigger time, and for 8 participants average wear time stopped on or before the last trigger time. Some participants (eg, participant 14) recharged the watch and put it on again, resulting in a median wear time much higher than the clock times of the longest episode.

The completion rates of watch questions varied. On average, twice daily questions were answered by 60% (15/26; morning) and 52% (14/26; afternoon) of participants, the once daily questions by 66% (17/26), and the weekly questions by 69% (18/26) of participants. The longer, monthly questionnaires that remained open for 1 week were answered by 89% (23/26) of participants.

**Figure 4:**
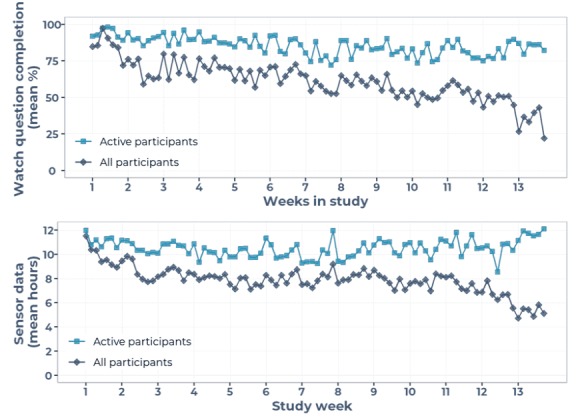
Engagement per study day, showing mean hours of sensor data (lower) and mean watch question completeness (upper). Dark blue diamonds correspond to median over all 26 participants; light blue squares correspond to median over all active participants.

The median watch question completion rates and hours of sensor data decreased over the study duration (dark blue diamonds in Figure 4). Engagement of *active* participants remained roughly constant through time (light blue squares in Figure 4).

### End-of-Study Survey: Participant Experiences

**Figure 5:**
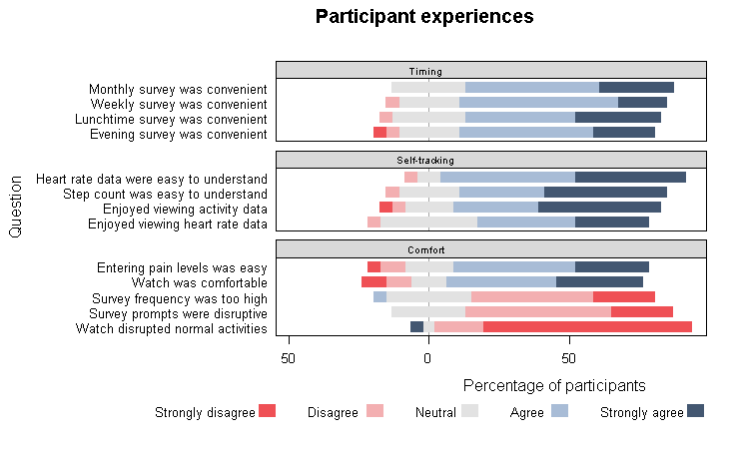
End-of-study study survey—Comfort, convenience of prompts, and self-tracking. Proportion of participants (100%=23) that chose a specific answer; bars to the right of zero reflect positive experiences.

A total of 23 participants completed the Web-based end-of-study questionnaire (Figure 5). Most participants found the watch comfortable, found it easy to enter pain levels on the watch, and found the timing of the various questions convenient. Only 1 participant found survey frequency too high, and 1 participant found the watch disrupted their normal activities.

### Participant Interviews

A total of 19 participants were interviewed at baseline, and 18 of these completed an end-of-study interview 3 months later (mean age 64 years; 10/19, 53% female). Analysis of the transcribed interviews identified themes around context, motivations, and expectations (section 1), interaction with the watch, experiences, and usability (section 2), and self-tracking (section 3).

#### Context, Motivations, and Expectations

The opportunity to learn more about the relationship between pain and activity was the primary motivating factor for the majority of interviewed participants (N=14). Some participants expected that participation would help them develop strategies to manage their pain better (Textbox 1, quotes 1.1 and 1.2). Others were motivated by the prospect of helping others and contributing toward improving knee OA research (Textbox 1, quotes 1.3 and 1.4).

**At baseline, most participants’ motivation was to learn about their condition, whereas some enrolled to contribute to research.**

1.1 I thought I might learn things from people...I might find out things that would help me. There was a degree of self-interest.

1.2 That's really why I'm taking part, to find out what I should do, what I shouldn’t do, what I...when to rest, when not to rest, when to be active, when not to be active. Anything that could help me find out about the condition and how to maybe alleviate it

1.3 I saw this, I thought, well if it’s anything that helps, even if it doesn’t help me personally now, if it helps in the long term, it can’t be a bad thing really, that was what attracted me.

1.4 I think if they can see some results from it, and it’s going to improve knowledge and so on. Some people might want to be motivated by a bit of money or something like that, but I think the vast majority would do it because it’s for the good of people, and hopefully improving the knowledge of people.

#### Interaction With the Watch: Experience and Usability

Although some participants expressed concerns in the preliminary interviews about successfully operating the smartwatch, all participants stated at follow-up that they found the watch easy to use (Textbox 2, quote 2.1). Participants did not consider answering the twice daily questions a burden. In fact, many participants suggested pain data should be collected more frequently, for example, also in the evening (Textbox 2, quote 2.2). Participants were enthusiastic about recording pain levels, and some suggested to add a “pain button” to record pain in real time (Textbox 2, quote 2.3). Participants explained that their engagement with the watch was affected by other activities. Sometimes they were too busy to answer the question at the trigger time, or they removed the watch for activities (eg, writing, gardening, and swimming) and forgot to put it back on. Battery life significantly influenced patterns of engagement, particularly for those participants who worked full days (Textbox 2, quote 2.4). Sometimes, participants missed the evening questions because of limited battery life. There appeared to be an expectation that the battery should last from early in the morning to late in the evening without recharging (at least 15 hours). Step count was automatically reset to 0 after recharging, frustrating some participants (see case study 2).

**Participant experience with smartwatch and usability (quotes from follow-up interview).**

2.1 It was easier than I thought really...When I came home, my husband went through it with me and I've been dead surprised how easy it was. I thought, I won't be able to do that… but I was surprised how easy I found it, yeah.

2.2 I think they could have asked a lot more, I think initially, I wouldn’t say I was frightened by it, but I was a bit intimidated, God, what’s this going to be like? When you got into it, and began to realise the sequences of questions, I think in a way it was a bit of a lost opportunity from your side, because I think there would have been the opportunity to ask quite a lot more.

2.3 I just felt I wanted to provide more information. I wanted to say that I’m in agony. So I think that maybe something that I could wear on that hand and I could say, right, that hurts now; that would be fine.

2.4 I think I struggled throughout with battery life and because I start work...I would be putting the watch on at about half past seven it didn’t always make it through to the end of the day when I got home, which would be like five or six. And so, I'd have to recharge it to start it, you know, to do the survey, which is often why I ended up missing because I'd just plug it in rather than do the survey...I must admit, in the end I was relieved to get rid of the watch because the battery was doing my head in at work, you know?

#### Self-Tracking

Participants stated that being involved in the study had helped them to focus more on their activity levels and challenge existing assumptions regarding their activity and pain (Textbox 3, quote 3.1). For example, 1 participant had previously avoided walking long distances as she associated walking with pain. Once she started tracking her steps, she became aware of how far she could walk without causing pain, which led to greater confidence and increased activity (Textbox 3, quote 3.2).

Not all participants found the step counter or heart rate monitor useful. Participants already interested in self-tracking had concerns regarding the accuracy of the watch as the watch data were different to their personal devices (Textbox 3, quote 3.3). As the majority of participants were primarily motivated by the opportunity to learn more about their condition, feedback was an important issue. Some participants mentioned that they would have preferred to be more active in analyzing their own data on a daily basis. However, irrespective of the fact that participants had doubts concerning the accuracy of the sensor data, the majority of participants remained in the study for the 3-month period.

**Participant experience with self-tracking and watch feedback (quotes from follow-up interview).**

3.1 It’s made me very aware on a daily basis of where I am, what things I’m doing...because you are actually charting progress, lack of progress and whatever that may be, that journey that you’re on. And the fact that you’re focusing three or four times a day; it really does say yeah I felt okay today...I just really enjoyed the experience and I felt I was getting something out of it.

3.2 If someone said to me, let’s go on a five mile hike, my absolute answer would be, no, my legs wouldn’t let me do it. What the watch and the app rather than the phone, has told me, is that I can do that, ‘cause I do that every day.

3.3 I don't think it was accurately recording footsteps. I have an app on my phone that does footsteps and I know that's pretty accurate because I've tested it against another app .I compared that against the watch and the watch was nowhere near.

### Case Studies

To illustrate how these themes interrelate to determine individual levels of engagement, we present 2 case studies: (1) a highly engaged participant despite no interest in self-tracking and (2) a participant that, in spite of interest in self-tracking, dropped out early. Each demonstrates how engagement results from a balance of the themes highlighted above, not always driven by the commonest themes.

#### Case Study 1—Highly Engaged

**Figure 6:**
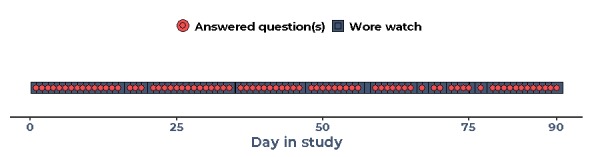
Engagement of case study 1 over 90 study days.

The participant was diagnosed with OA over 10 years ago. He wore the watch for 88 days and answered on average 73% (79/249 days; Figure 6) of the watch questions. He had no previous experience with self-tracking or wearables, meaning his high engagement in the study was not explained by any prestudy interest in self-tracking. In fact, at baseline, he considered self-tracking to be “narcissistic” (Textbox 4, quote 4.1).

The participant had no expectations that the study would benefit him personally. His reasons to participate were altruistic in nature as he emphasized that while the study may not benefit him, it could benefit others (Textbox 4, quote 4.2).

He occasionally missed questions on some days. He explained this was because of his daily routine, as he sometimes worked late, and he would forget to recharge the battery. He reported that he was glad to finish the study, as he did not particularly like the look of the watch (Textbox 4, quote 4.3).

Irrespective of a negative attitude toward self-tracking and the look of the watch, the participant had the highest level of engagement in the study. This paradox may be explained by the fact this participant had no expectations of personal benefit from participating in the study; hence, negative aspects of the watch did not disappoint him.

**Case study 1: experiences of high engager without previous self-tracking experience (4.1 and 4.2 from baseline interview; 4.3 from follow-up interview).**

4.1 I’ve no desire, okay I’m sure there are uses of smartwatches but it doesn’t have place in my way of life and I can't see how it would do...Well, its over-indulgence, over focus on your own...

4.2 Whatever you learn will somewhere down the line, it'll be used. (...) I just wanted to find out what it’s about, and also maybe help other people who might get it in the future.

4.3 I think a couple of days I forgot to wear it and I think it was probably I had to leave it in my slipper charging so that I wouldn't forget it. And I was glad when I didn't have to wear it anymore...wearing the watch was not a pleasure but we didn't sign up to look cool. Well it’s a big ugly heavy rubber watch, there’s nothing particularly aesthetic about it.

#### Case Study 2—Early Study Withdrawal

**Figure 7:**
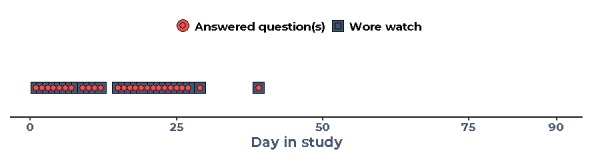
Engagement of case study 2 over 90 study days.

Some participants were highly engaged for the first part of the study but then started using the watch significantly less, or in some cases not at all. This participant was diagnosed with OA within recent years. She wore the watch for 27 days, answering 79% (26/88 days; Figure 7) of watch questions on average. At the beginning of the study, she was fully engaged and answered all the daily questions. In contrast to case study 1, this participant was motivated to participate in the study to learn more about how to deal with her pain and potentially avoid having surgery (Textbox 5, quote 5.1). She enjoyed the process of answering the daily questions at the beginning of the study as focusing on her pain challenged her previous assumptions regarding activity levels and pain (Textbox 5, quote 5.2). She had not done self-tracking before the study, but she did enjoy this at the beginning.

Over time, she increasingly became skeptical and frustrated with the technical aspects of the watch: the step counter reset when she recharged it, and she was not convinced of its accuracy (Textbox 5, quote 5.3). She was also concerned that nonambulatory triggers for her pain, such as standing and bending, were not captured by the watch (Textbox 5, quote 5.4). She found the size of the watch to be too big, and she mentioned that it got in her way when writing (Textbox 5, quote 5.5).

After this series of disappointments, the participant experienced technical problems. The watch would frequently turn itself off and eventually stopped recharging. The study team offered to send a new watch, but because of her overall frustrations with the watch, she declined this (Textbox 5, quote 5.6).

In conclusion, concerns about the data quality and problems with the size of the watch caused disengagement. Owing to the sequence of disappointments by the time the watch broke, she could not be persuaded to remain in the study.

**Case study 2: experiences of a participant that enjoyed learning from the self-tracking but dropped out after a series of unmet expectations (quote 5.1 from baseline interview; 5.2 to 5.6 from follow-up interview).**

5.1 It’s the idea of being better informed about what’s causing the pain, and actually if anybody can find a way of helping with the pain, without having surgery, then that’s got to be a good thing.’

5.2 “It’s been brilliant because I was aware, for example, that some of my worst times can be when I’m at work, and I can be sitting there, and I stand up and I’m in agony...So whilst I can’t see that I’ve contributed anything to kind of findings, if you like, from a personal point of view it’s been good because I think it has helped me understand what hurts and what doesn’t hurt, and I’m not as idle as I think. So that’s quite good.”

5.3 I just had some questions about the reliability; and although they said that it was still picking up steps, I didn’t have confidence that it was providing an accurate reflection of the steps that I’d done I was frustrated because I don’t think I ever had a complete day where I could be confident that actually I had walked quite a long way, and I thought I’d definitely walked more than the steps that it was showing, or it was cleared off, I couldn’t ever tell how many I’d done.

5.4 It just raises lots of questions in my mind about, what do they really know about what I’ve been doing? ‘Cause I was outside yesterday and I probably didn’t do that many steps. But actually that’s irrelevant, it’s the fact that I’m bending. That is much more relevant to my knee pain...And the other thing is, standing. Just standing is a nightmare… And that wouldn’t be captured, because I wouldn’t have done any steps at all, ‘cause literally, I was just standing there. And for me, that would have been a million times worse than walking loads of steps on the flat.”

5.5 I can’t write with it on, it was far too uncomfortable, the watch face is too big. And again I suppose that’s a, you know, women generally have smaller watches, we’re not used to it; but if I was writing it would pinch my skin, otherwise if I didn’t have it reasonably tight it would flop around and the face would end up there, and then there’d be that bone there and I just couldn’t get away with it.

5.6 I’d had a few problems with it; and then I had a little spell where it seemed to be better, so although I’d contacted the team and said I was having these problems, it then picked up; and then it just got really bad, so I contacted them again and said, I’m just not getting away with it because of...you know, because I always thought ‘’has that captured what I’ve said?’, or if it was on charge the question didn’t come back again. So although the team said you could still do it later, and they said that steps would still be captured, I didn’t think it was reliable.”

The case studies show that expectation of personal gain or learning about someone’s condition alone does not explain engagement. Case study 1 shows that participants motivated by altruism may stay engaged even if they have little interest in self-tracking. Case study 2 shows that those with high interest in self-tracking also may have higher expectations, which, if not met, can be a reason for disengagement.

## Discussion

### Principal Findings

This study succeeded in collecting frequent sensor data and patient-reported outcomes using cellular smartwatches for 90 days. Participants wore their smartwatch on most days, with engagement declining most notably after week 12 as participants approached the study end date. Among participants who remained active, data completion remained high, and neither watch wear time nor completion rate of watch questions declined significantly over time. Most participants joined the study to learn more about the link between their pain and activity, in line with known benefits from symptom tracking [24]. They found the watch app technically easy to use even though most had no previous experience with self-tracking. Several participants found the watch somewhat big or cumbersome. Participant interviews showed that the main barriers to wearing the watch were battery life limitations, technical problems, and unfulfilled expectations of, or doubts about, the watch performance. The first case study illustrated that an interest in self-tracking (one of the commonest motivators) is not an essential requirement for high engagement. The second case study showed that being interested in self-tracking does not automatically lead to high engagement: this participant dropped out after recurrent small disappointments where the watch did not meet her expectations.

### Strengths and Limitations

The study has a number of strengths. To our knowledge, it is the first to develop an app to collect both patient-reported outcomes and sensor data from this new generation of consumer cellular smartwatches. The combination of quantitative methods and qualitative methods provides important insights into motivations and barriers for participant engagement with the new technology. The case studies show how these motivations and barriers are weighed against one another. Although the app is not publicly available, lessons learnt about engagement are transferable to future consumer cellular smartwatch studies.

A limitation of this study is the small, self-selected sample. Participants had volunteered for the study, which means that they may have been more motivated than the nonvolunteering population with OA, resulting in higher engagement. Second, we may have underestimated watch wear time. We could not directly record wear time but instead defined this as “minutes of sensor data received.” This definition excludes time that a participant wore the watch, but it was out of battery or out of internal memory. Third, we cannot draw conclusions about data collection for longer periods of time, as our feasibility study was limited to a 3-month period.

### Comparison With Prior Work

High attrition rates are often a characteristic of mobile health studies [22] and even of activity trackers for personal use [25]. Until the last week of our study, attrition was relatively low. In a 6-week study of Fitbit activity trackers, most participants dropped out (75% attrition after 4 weeks, compared with 4% in our study) [26]. Despite asking participants to report symptoms 4 or 5 times per day, we retained higher completion rates than studies that requested information fewer times per day to OA patients [10,11] or other patient groups [22,27,28]. The possible burden of higher number of questions may have been offset by the speed of data entry per question: responding on a wrist-worn device took less than 10 seconds, compared with taking out a device or diary in other studies. The workload and time required to enter data are known to influence attrition [22], but it remains uncertain where the balance lies between frequency of entry and duration required per entry. A total of 12 participants stopped wearing the watch in the last week of the study but before their end date. Enrollment was staggered over 12 days, but participants received instructions for returning the watch on the day that the first participants had completed 90 days. This may have led to possible confusion for late enrollers, thinking the study had already ended for them.

Barriers and motivators that were identified in the interviews largely correspond with previous research. Our participants were primarily motivated to learn about their condition, a common motivation to engage with digital health apps [28]. Most barriers to engagement have also been described in other studies: forgetting to charge or put on the watch [26], physical design and aesthetics [26], issues with (expectations of) data accuracy [26,28], and preferring a competing intervention [22].

Receiving feedback from a digital health app has previously been identified as a motivator [28]. In our study, participants perceived wearing the watch as beneficial, even though they did not receive decision support and could not look back into previous pain or step count values. Many stated that using the watch still led to a better understanding of the relation between their pain and activity. We did not find “lack of previous experience with digital devices and health tracking” [28] as an important barrier to engagement, possibly because participants with limited digital literacy also found the watch and our app intuitive and easy to use.

This study focused on usage of consumer cellular smartwatches for research only, rather than for self-management or clinical care. Self-tracking using consumer devices has advantages for self-management such as giving participants a better understanding of their condition (an advantage also observed in this study) and identifying triggers [24]. Our app did not display visual feedback about recently tracked symptoms, which may have limited such benefits. Self-tracking also has the potential to transform clinical consultations by providing a clearer picture of symptoms while at home, improving shared decision making [29]. Integrating data from smartwatches into electronic health records in the future may well deliver similar advantages.

### Recommendations For Future Studies

Future studies may increase engagement in a number of ways. Our interviews indicate that unrealistic expectations of watch performance (eg, battery life) and doubts about accuracy of the device or ability of researchers to derive relevant metrics caused participants to disengage. Better participant information upon enrollment might mitigate this source of attrition. Visualization of the participants’ own data may increase engagement further, especially given the primary motivator of participants wanting to understand better their relationship between physical activity and pain. However, such a change needs to be balanced against concerns that feedback may influence subsequent reporting. Improvements in the technology, including longer battery life and lighter, more comfortable watches, may further reduce attrition.

### Conclusions

This study suggests that it is feasible to use cellular smartwatches for collection of patient-reported outcomes 4 or more times per day alongside continuous sensor data collection. Indeed, participants felt self-reported data collection could be even more frequent than the 4 or 5 times per day in this study. Learning about symptoms was a prime motivator to use the watch, even though most participants had never self-tracked before. Technical issues rather than participant attitudes more commonly limited engagement with the smartwatches. Overall, cellular smartwatches were an acceptable and feasible new data collection tool to support health research.

## Appendix

Multimedia Appendix 1 Overview of interview topic guides.

## Abbreviations

KOALAP: Knee OsteoArthritis: Linking Activity and Pain
OA: osteoarthritis
