# Supplementary material for: Engagement and Participant Experiences With Consumer Smartwatches for Health Research: Longitudinal, Observational Feasibility Study
Source: JMIR Mhealth Uhealth. 2020 Jan 29;8(1):e14368. doi: 10.2196/14368 (PMC7016619; doi:10.2196/14368)
Supplement: Multimedia Appendix 1 [file mhealth_v8i1e14368_app1.pdf]

Overview of interview topic guides

Appendix to: Beukenhorst *et al.* Engagement and Participant Experiences With Consumer Smartwatches for Health Research: Longitudinal, Observational Feasibility Study**.** *JMIR Mhealth Uhealth* 2020; 8(1); e14368.

OA = osteoarthritis

Baseline interview

- **Health and existing long-term conditions**: overall health, timing of OA diagnosis, previous knowledge of OA, comorbidities (especially other musculoskeletal conditions or mental health conditions) and other health problems
- **Management of OA:** healthcare (medication, operations, joint replacements), lifestyle changes since diagnosis (diet, exercise, social support groups, changes to home), reasons for (not making) lifestyle changes, barriers to lifestyle changes
- **Impact of OA in life**: on day-to-day activities, emotional wellbeing, mood, coping, social life, relationships with family, friends or work colleagues?
- **Views and opinions on using a smart watch app to collect KOALAP data**: views on storing and sharing health data, on collecting information about peoples' position and movements, concerns, perceived benefits, expected effects on own OA management
- **Previous experience of using smartphone, other devices and apps**: usage or ownership of smartphone, tablet or computer, frequency of use, reason of (non-)usage, experience with health-related apps
- **First impression of the watch**: expectations of usage, daily symptom monitoring and daily wearing the watch

Follow-up interview

- **Recent OA symptoms**: overall health, OA symptoms since last interview, perceived experience of pain-activity relationship
- **Experience with self-reporting functionalities**: experience, ease of use, difficulty of use, useful aspects, unuseful aspects, experience of self-reporting symptoms, frequency of completion, reasons for (not) completing surveys, experience with questions, suggestions for changes to questions
- **Experience of passive monitoring**: experience being tracked, any differences with expectations, what did the watch track, and was this a true reflection of your movements, impact on movements, experience wearing the watch, frequency and duration of (not) wearing the watch
- **Smartwatch and self-management**: impact on view of OA symptoms, impact on frequency of thinking about OA symptoms, impact on managing changing symptoms, usage of activity data on smartwatch and graphs in smartphone app
- **Usability**: daily reminders, battery life, charging, cellular connection
- **Prolonged usage**: willingness to wear the watch for a year (+ reasons), reasons why some people won't be willing to use the watch long term, reasons/incentives to encourag long-term involvement in others
